# Dataset on georeferenced and tagged photographs for ecosystem services assessment, Ebro Delta, N-E Spain

**DOI:** 10.1016/j.dib.2020.105178

**Published:** 2020-01-25

**Authors:** David Serrano Giné, María Yolanda Pérez Albert, Aitor Àvila Callau, Joan Jurado Rota

**Affiliations:** Department of Geography, GRATET, Universitat Rovira i Virgili, c/ Joanot Martorell, 15, 43480, Vila-seca, Spain

**Keywords:** Volunteered geographic information (VGI), Landscape analysis, Image content, Protected areas, Wikiloc

## Abstract

A georeferenced and tagged dataset of photographs is presented. Over 2000 photographs from the Ebro Delta Natural Park, N-E Spain, have been treated. Raw data come from Wikiloc, a Volunteered Geographic Information source, and have been cleansed and systematized. The photographs have been classified according to their image content. An automatic first analysis was performed using 8-bit software. For uncertain tags, a second supervised analysis was performed. Classification into eight types and thirty-seven subtypes was conducted by considering landscape and social reactions. Data have been treated with the ArcGis 10.2. Geographic Information System. This dataset is useful for understanding ecosystem services by means of users' photographs.

**Table:** Specifications Table

Subject	*Geography*
Specific subject area	*Volunteered Geographic Information (VGI), ecosystem services, protected areas, image content, landscape analysis*
Type of data	*Table, vector spatial data*
How data were acquired	*Volunteered Geographic Information, Geographic Information System, 8-bit software*
Data format	*Raw, Filtered, .shp files*
Parameters for data collection	*Photographs from Wikiloc website were acquired for the area under study for the period 2006–2016. Data mining procedures were used to download data.*
Description of data collection	*Photographs were systematized and classified via automatic (8-bit software) and supervised procedures; attribute tables were constructed and joined using ArcGis 10.2.*
Data source location	*Ebro Delta (40º 43′ 05 N, 0º 41′ 18E WGS 1984), Catalonia, N-E Spain*
Data accessibility	Repository name: *Mendeley* Data identification number: *1* Direct URL to data: https://doi.org/10.17632/3ny5krr9k2.1
Related research article	*A. Callau Àvila, M. Y. Pérez Albert, J. Jurado Rota, D. Serrano Giné, Landscape characterization using photographs from crowdsourced platforms: content analysis of social media photographs, Open Geosci. 11 (2019) 558–571.* *DOI:*https://doi.org/10.1515/geo-2019-0046

**Table:** 

**Value of the Data** • The data provide a set of georeferenced photographs classified according to image content into landscape types and users' social reactions. • The dataset is useful for managers and planners designing strategies for public use in protected areas according to users' preferences. • The dataset serves as a reference for comparative studies on users' preferences and public use in protected areas. • The dataset helps to explain users' preferences via photographs in protected areas. • The dataset is a reference on the automatic content analysis of photographs for ecosystem services assessment.

## 1 Data Description

The dataset is organized in an ESRI shapefile that maps and provides thematic information on photographs. Raw data come from Wikiloc [1], a crowd sourced sports website popular with visitors to the area. The dataset comprises 2131 photographs uploaded between 2006 and 2016. These photographs are of Volunteered Geographic Information type and have been cleansed and systematized for better use and understanding.

The photographs were classified using image content software. Supervised classification was conducted when automatic classification was unclear. The photographs were classified by eight types and thirty-seven subtypes.

**Fig. 1:**
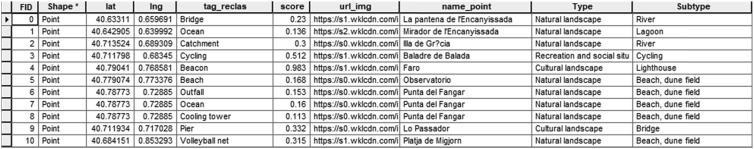
Screengrab of the dataset.

The resulting attribute table comprises latitude and longitude coordinates, a descriptive tag, a score for the probability of the photograph being true (only scores equal to or above 0.10 were considered), the photograph's URL, the name of the photograph (recorded by the user who uploaded it), and the photograph type and subtype. Fig. 1 shows a screengrab of the dataset as it appears on the original.shp file.

**Fig. 2:**
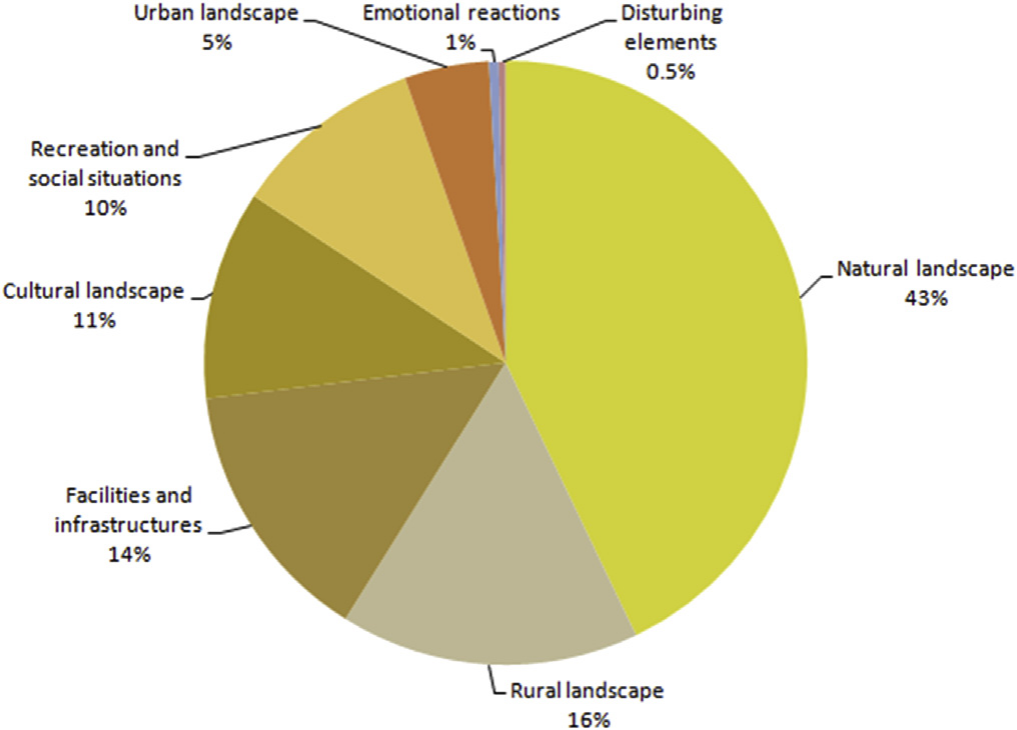
Percentage of photographs per landscape type.

**Fig. 3:**
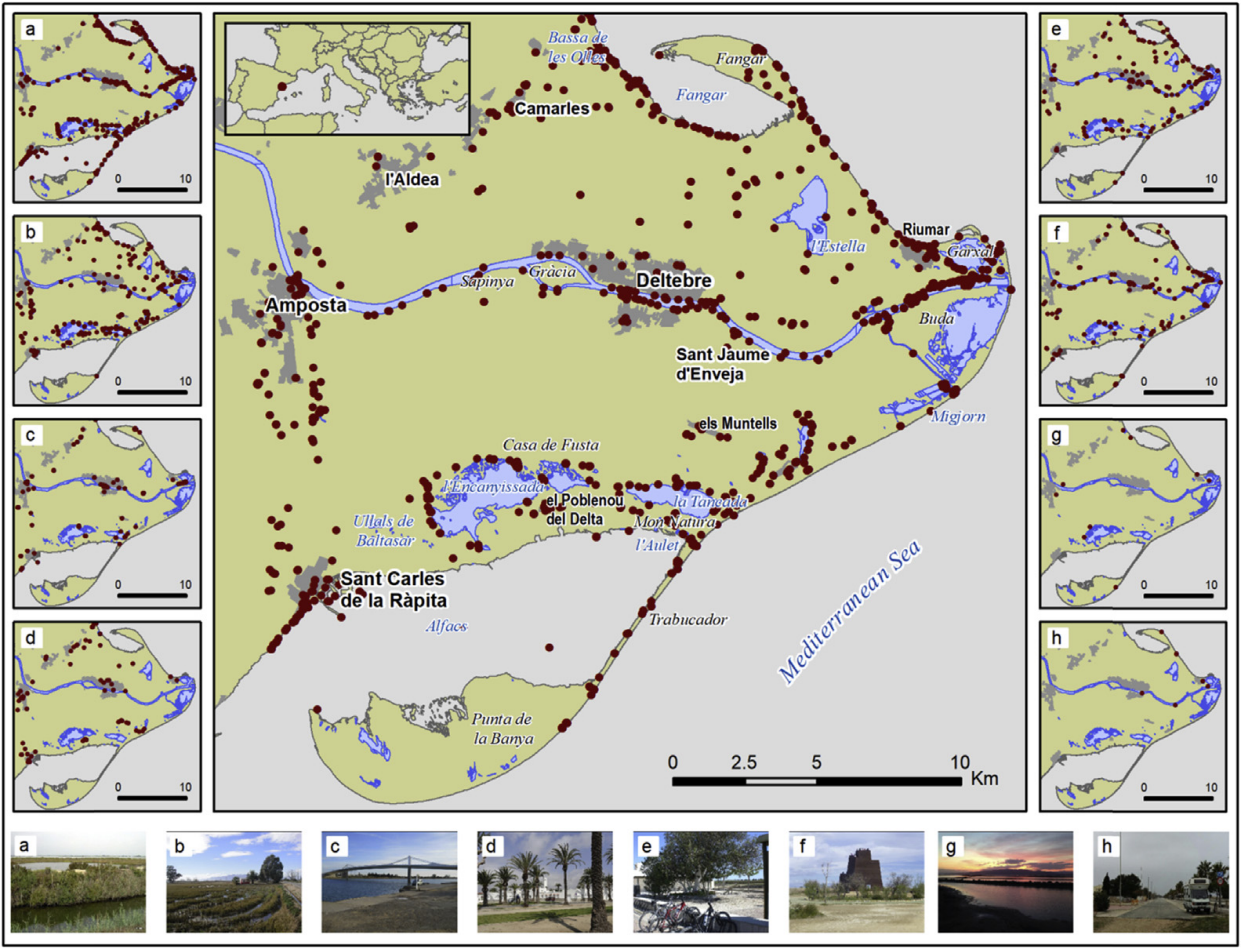
Photographs in the database. Main map: general overview; a: natural landscape; b: rural landscape; c: cultural landscape; d: urban landscape; e: recreation and social situations; f: facilities and infrastructures; g: emotional reactions; h: disturbing elements.

Information about the percentage of photographs for each landscape type can be found in Fig. 2. The main map in Fig. 3 shows the location of all the photographs analyzed, and a set of eight auxiliary maps shows the location of the photographs according to their landscape type; a representative photograph for each landscape type is also included (Fig. 3).

**Fig. 4:**
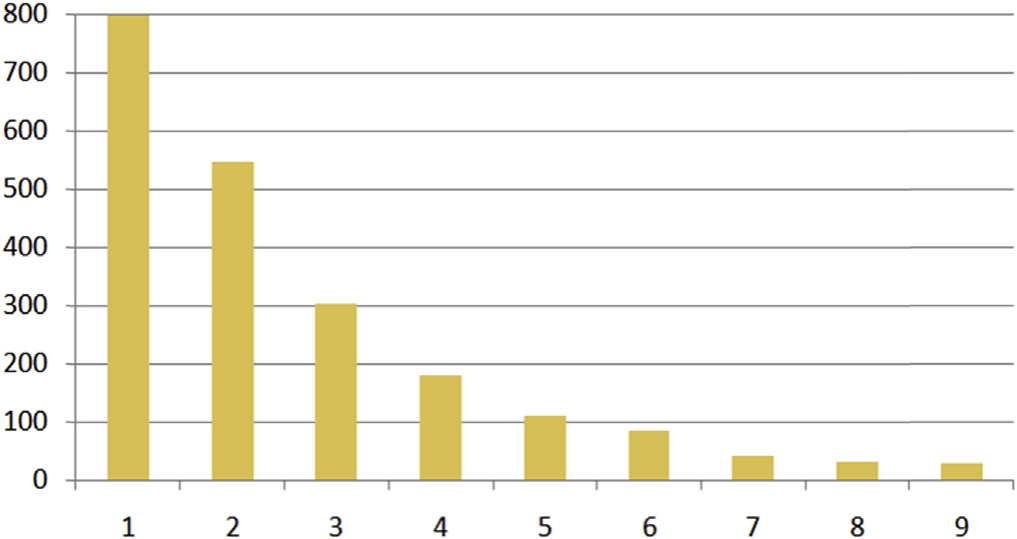
Confidence scores for the tags to be true.

**Fig. 5:**
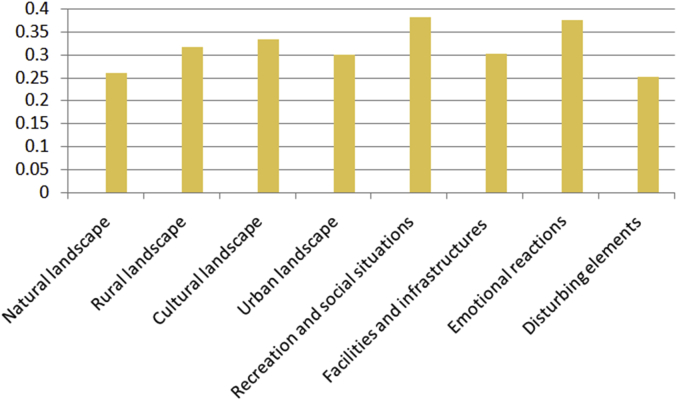
Mean confidence scores for type of photograph.

The number of photographs and their confidence scores for the tags to be true is showed in Fig. 4, and the mean confidence scores for each type of photograph is reported in Fig. 5.

## 2 Experimental design, materials, and methods

We analysed, classified and characterized photographs from Volunteered Geographic Information data in the Ebro Delta, a protected area in north-east Spain, taken between 2006 and 2016. Wikiloc, a sports web-based data-sharing platform popular with users in the area under study [2], was used as a data source. The experimental design comprised analysis of photographs freely uploaded by users of the Wikiloc platform.

The photographs were treated in two steps. First, they were systematically classified according to their image content [3]. 8-bit software was used to tag the images using descriptive words. A descriptive tag and a score for the probability of their being true were added. This software generates a score for the probability of their being true that ranges from 0.01 to 0.99. Any label whose score was above 0.10 was considered reliable. Supervised classification was conducted for some photographs that were difficult to tag automatically [4]. Spatial output for each photograph was conducted using ArcGis 10.2, and projected in European Terrestrial Reference System 1989 datum, UTM projection zone 31 N. Attribute tables were also constructed and joined using ArcGis 10.2.

The photographs were acquired using data mining procedures and, as they come from Volunteered Geographic Information, they are provided freely by users and have no privacy issues.

The descriptive tags were then classified according to their meaning, with landscape and social reactions considered as references. The final classification comprises eight types and thirty-seven subtypes. This was inspired by Ref. [5], and more detail can be found in the associated publication [7]. Four of the eight types typify the landscape while four typify social reactions or interests. The types are: Natural landscape, Rural landscape, Cultural landscape, Urban landscape, Recreation and social situations, Facilities and infrastructures, Emotional reactions, and Disturbing elements.

The photographs reveal users' preferences on landscape and social reactions to their place-experience. Some photographs are easy to understand from an ecosystem services point of view, while others seem meaningful only to the users who uploaded them. The total number of valid photographs was 2,131, three quarters of which were of landscapes. These were mainly natural landscapes (43.1%), followed by rural landscapes (16%) and cultural landscapes (11%) (Fig. 2).

The spatial distribution of the photographs reveals clusters of images [6]. More information about the photographs and their subsequent analysis can be found in the associated publication [7] (Fig. 3).

8-bit software considers that any tag with a score of over 0.1 is reliable. Almost two fifths of the tags have a confidence score of between 0.1 and 0.19, almost a third have a confidence score of between 0.2 and 0.29, and 14% have a confidence score of between 0.3 and 0.39 (Fig. 4). With regard to photograph types, the mean values of confidence scores are highest for Recreation and social situations and for Emotional reactions (0.38), though extreme values appear on any type of photograph: for instance, 50% of photographs with a score equal to or above 0.90 belong to Cultural landscapes, while 14% belong to Natural landscapes and 14% belong to Facilities and infrastructures (Fig. 5).
